# Fecal Microbiome Alterations in Colorectal Cancer: A Systematic Review of Compositional Changes and Microbial Biomarkers

**DOI:** 10.1002/mbo3.70326

**Published:** 2026-06-17

**Authors:** Parvin Askari, Shirin Dashtbin, Tahereh Navidifar, Leila Dadgar‐Zankbar, Arezoo Asadi, Mahsa Ghamari, Parisa Najafi, Shabnam Zeighamy Alamdary, Roghayeh Afifirad, Roya Ghanavati, Atieh Darbandi

**Affiliations:** ^1^ Infectious Diseases Research Center Birjand University of Medical Sciences Birjand Iran; ^2^ Department of Microbiology, School of Medicine Iran University of Medical Sciences Tehran Iran; ^3^ Microbial Biotechnology Research Centre Iran University of Medical Sciences Tehran Iran; ^4^ Department of Basic Sciences Shoushtar Faculty of Medical Sciences Shoushtar Iran; ^5^ Endocrine Research Center, Institute of Endocrinology and Metabolism Iran University of Medical Sciences Tehran Iran; ^6^ Department of Pathobiology, School of Public Health Tehran University of Medical Sciences Tehran Iran; ^7^ Faculty of Sports and Exercise Science University Malaya Kuala Lumpur Malaysia; ^8^ Department of Microbiology, School of Medicine Tehran University of Medical Sciences Tehran Iran; ^9^ Department of Basic Sciences Behbahan Faculty of Medical Sciences Behbahan Iran; ^10^ Molecular Microbiology Research Center Shahed University Tehran Iran

**Keywords:** bacteria, biomarker, colorectal cancer, feces, gut, microbiome

## Abstract

Colorectal cancer (CRC) is one of the most common types of cancer worldwide, and the gut microbiome plays a crucial role in its development. In the study, we examine the variation in gut microbial community composition among individuals diagnosed with CRC based on human fecal samples. A systematic search of online databases, including MEDLINE (PubMed), Web of Science, Embase, and Scopus up to March 2026, following the requirements outlined in the PRISMA guideline. The search strategy was based on a combination of keywords, including “colorectal cancer,” “gut microbiome”, and “feces.” The study analyzed 43 research articles on colorectal cancer microbiome. Most investigations utilized culture‐independent techniques, revealing variations in microbial profiles between colorectal cancer cases and healthy controls. *Fusobacterium* and *Porphyromonas* emerged as potential colorectal cancer biomarkers, while multi‐bacteria predictive models showed promise in enhancing colorectal cancer detection sensitivity and specificity. In this review, we will explore how advanced sequencing techniques have the potential to complement current non‐invasive methods for early diagnosis and prevention of colorectal cancer. This includes conducting studies with robust statistical power and consistent, replicable methodologies, taking into consideration host factors, and performing external validation of predictive models.

## Graphical abstract

Gut microbiome plays a crucial role in colorectal cancer development. The advanced sequencing techniques have the potential to complement current non‐invasive methods for early diagnosis and prevention of colorectal cancer. This includes conducting studies with robust statistical power and consistent, replicable methodologies, taking into consideration host factors, and performing external validation of predictive models.

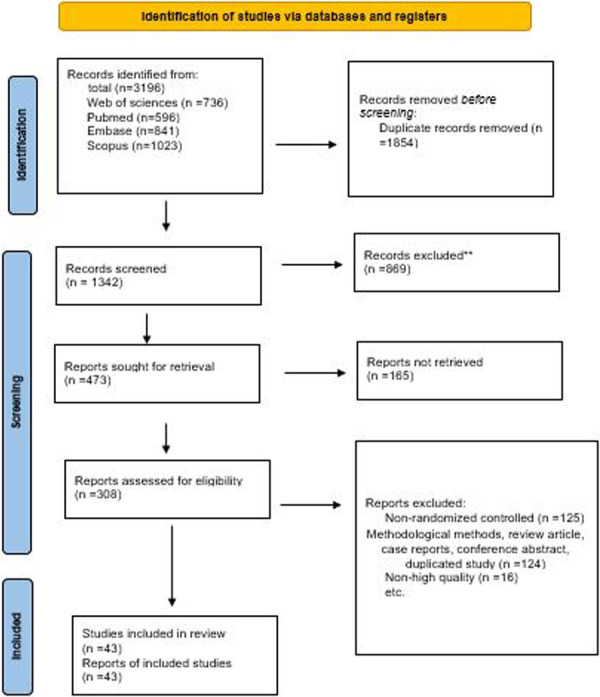

## 1 Background

Colorectal cancer (CRC) is the third most common cancer worldwide, following lung and prostate cancer in males. It is also the second most common malignancy after breast cancer in females (Ferlay et al. 2015). In the early stages, CRC often displays no symptoms, and the majority of cases develop slowly from adenomatous precursors (Ferlay et al. 2015; Pawa et al. 2011). Unfortunately, most cases are diagnosed when the cancer has reached advanced and uncontrollable stages. Therefore, early detection is crucial for improving the survival rates of CRC patients. Traditional screening methods such as barium enema, colonoscopy, and sigmoidoscopy are uncomfortable, invasive, time‐consuming, and expensive (Sung et al. 2015). Consequently, there is a pressing need to identify and explore new biomarkers and reliable diagnostic methods for CRC. Studying the composition of the intestinal microbiome in CRC patients can provide new insights into tumor screening methods. Recent studies have suggested that microbiota profiles determined by high‐throughput sequencing could be effective in predicting CRC (Zeller et al. 2014). The gut microbiota may play a significant role in tumor initiation and progression, as the incidence of cancer in the large intestine is approximately 12 times higher than that in the small intestine, which is attributed to a greater bacterial density in the colon (10^12^ cells per ml) compared to the small intestine (10^2^ cells per ml) (Brennan and Garrett 2016). Consequently, any imbalance in the healthy gut microflora or dysbiosis may result in various diseases such as diabetes, obesity, metabolic syndrome, inflammatory bowel disease, irritable bowel syndrome, celiac disease, and CRC (Rezasoltani et al. 2017). The interplay between gene methylation and gut microbiota in CRC has also been identified. Gut bacteria can directly influence DNA replication, transcription, repair systems, RNA splicing, and chromatin remodeling (Lee 2011). Recent studies suggest that dysbiosis (changes in microbial diversity and microbial taxa) in the gut microbiota can disrupt microbial homeostasis and promote the proliferation of specific microbes, thereby contributing to CRC development through inflammation, damage to the gut barrier, and production of harmful metabolites (Hofseth et al. 2020; Ghanavati et al. 2020a). Other human diseases, such as obesity, type II diabetes, and inflammatory bowel diseases are also known to be significantly influenced by the gut microbiota (Crosbie et al. 2018; Parisa et al. 2020; Ghanavati et al. 2020b). Several bacteria have been associated with CRC, including *Peptostreptococcus anaerobius* (Long et al. 2019), *Bacteroides fragilis* and a strain of *Escherichia coli*, *Streptococcus bovis* (Boleij et al. 2009), *Clostridium septicum (*Seder et al. 2009 *)*, and *Fusobacterium nucleatum* (Castellarin et al. 2012). Recent metagenomics‐based studies have found that the guts of CRC patients contain enriched populations of *Parvimonas micra*, *Solobacterium moorei*, *Fusobacterium nucleatum*, and *Peptostreptococcus stomatis (*Tjalsma et al. 2012 *)*. Enterotoxigenic *Bacteroides fragilis* has been found in higher concentrations in the feces and colonic mucosa of CRC patients (Montalban‐Arques and Scharl 2019). The dysbiosis of the microbiota may contribute to the production of oncometabolites and tumor suppressor metabolites, which have adverse impacts on the immune system and genotoxicity (Bultman 2017). Dysbiosis can generate a variety of metabolites that play significant roles in the development of CRC, including short‐chain fatty acids (SCFAs) such as lactic acid, acetic acid, and propionic acid, secondary bile acids, colibactin, trimethylamine/trimethylamine‐N‐oxide (TAM/TMAO), and various other compounds (Alhhazmi et al. 2023). However, the exact mechanisms by which the human gut microbiota and these metabolites contribute to the development of CRC are still unclear. Therefore, understanding the roles played by the microbiome and the metabolome in the pathogenesis of CRC is crucial (Genua et al. 2021).

Understanding the factors that affect the composition of the microbial community in the gut is essential for developing treatments for CRC. In this systematic review, we examine the variation in gut microbial community composition among individuals diagnosed with CRC based on human fecal samples.

## 2 Methods

This systematic review was conducted according to the Preferred Reporting Items for Systematic Reviews and Meta‐Analysis (PRISMA) guidelines. Our systematic review has been registered in PROSPERO (CRD42024534857).

### 2.1 Search Strategy and Data Collection

Studies focusing on gut microbiome metabolites in CRC were identified through a systematic search of online databases, including MEDLINE (PubMed), Web of Science, Embase, and Scopus up to March 2026. The following search syntax was used to search PubMed and other databases. The search strategy was based on the following combination: “colorectal cancer,” “gut microbiome,” and “feces.” The logical operators “AND” (or the equivalent operator for the databases) were used to combine all descriptors to improve the results. Synonyms were identified using Mesh Terms, Emtree and the free text method and were selected after importing the results of a systematic online database search into EndNote21 (Thomson Reuters, New York, NY, USA) and removing duplicates. To avoid bias, two authors (TN and PN) independently searched and analyzed the relevant publications. The third author (AD) investigated any discrepancies and reached a conclusion.

### 2.2 Inclusion and Exclusion Criteria

**Figure 1:**
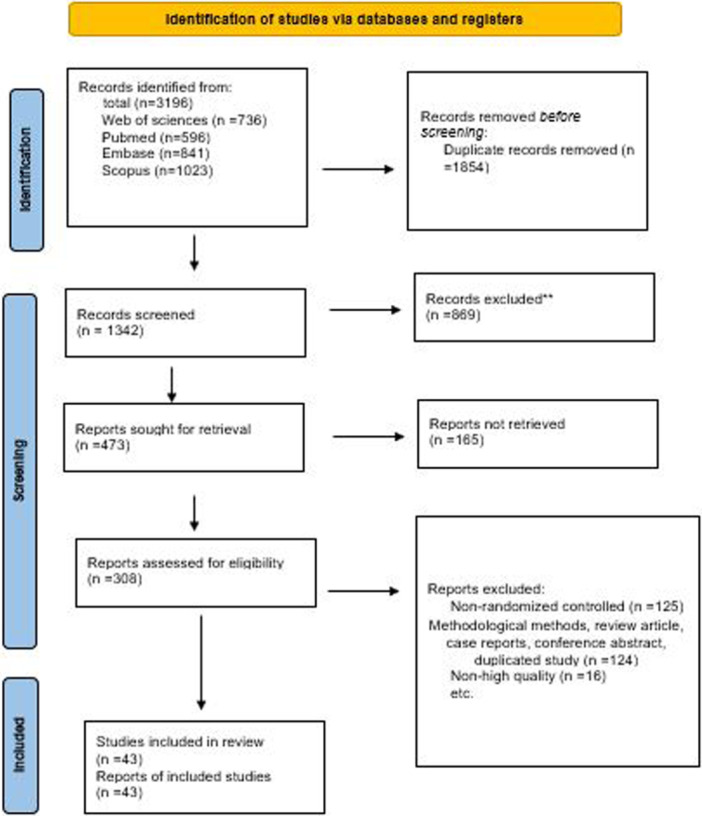
Flow chart of study selection for inclusion in the systematic review and meta‐analysis.

Figure 1 summarizes the exploratory methods outlined in the article. The eligibility criteria for inclusion of articles in this systematic review encompassed case‐control, observational, and randomized clinical trials involving adults or children with defined outcomes between 2010 and 2026, with high or moderate quality and full English text available. Additionally, the included articles were required to present results on the gut microbiome of feces in at least two groups: individuals diagnosed with CRC and colorectal‐adenoma‐ and CRC‐free subjects (referred to as “controls” in this article). Exclusion criteria were as follows: (1) animal studies; (2) data from duplicate reports, comments, notes, opinion pieces, methodological reports, conference abstracts, editorials, meta‐analyses, systematic reviews, or narrative reviews., (3) Articles that were not available or lacked full text; (4) Failure to access full‐text articles despite multiple attempts.

### 2.3 Data Extraction and Study Characteristics

Data were extracted separately by two reviewers, and discrepancies were resolved by building consensus. The studies were meticulously selected based on the following criteria: population, intervention, comparator, outcomes, and study designs.

#### 2.3.1 Population

Original articles that provided sufficient data on the fecal microbiome in patients with CRC were included in this study.

#### 2.3.2 Intervention

This review does not evaluate the effects of intervention measures on CRC, as this falls outside its scope. The primary objective is to assess the impact of the fecal microbiome in patients with CRC that may pose a risk to individuals.

#### 2.3.3 Comparator

The fecal microbiome of healthy individuals (without CRC) was considered as the comparator.

#### 2.3.4 Outcomes

The association between the fecal microbiome and CRC was investigated. For this reason, the following data were extracted from each study: (a) publication characteristics (first author, year, country, study design); (b) participant characteristics (sample size, patient age, gender); and (c) gut microbiome identified in fecal samples of CRC patients; (d) gut microbiome identified in fecal samples of healthy subjects; and (e) primary outcomes.

### 2.4 Quality Assessment and Publication Bias

The quality of the references was assessed using the Joanna Briggs Institute (JBI; The Joanna Briggs Institute, 2014). Studies underwent a quality assessment where each component was rated as “yes,” “no,” “unclear,” or “not applicable.” Subsequently, each study was assigned a score. In cases of disagreement, a third reviewer made the final decision.

## 3 Results

### 3.1 Study Characteristics

A total of 43 studies were included in the final analysis, and their main characteristics are summarized in Table 1.

**Table 1:** Characteristics of studies included in the systematic review.

No	First author/pub. year/Country(ref)	Data collection years	Study participants (males) Age mean (mean± median)			Stool collection	Temp. stool storage	Antibiotic use before stool sample	Database used for taxonomy assignment	Microbiome analysis method	Groups compared for bacterial differences	Statistical analysis for bacterial differences CRC
			CRC	Adenoma	Healthy							
1	Allali, 2018, Morocco (Allali et al. 2018)	October 2013 ‐December 2013	11 (52.8±54)	—	12(49.3±46)	Self‐sampled	−80°C	Not in 3 months	KEGG	V1–V216S rRNA	October 2013 ‐December 2013	11 (52.8±54)
2	Baxter, 2016, USA (Baxter et al. 2016)	—	120 (68) 63±12.9	198 (118) 63.3±11.5	172 (61) 54±9.9	Whole evacuated stool	−80°C	N. D	RDP	V4 16S rRNA	—	120 (68) 63±12.9
3	Yuan, 2021, China (Yuan et al. 2021)	—	226	—	156	Self‐sampled	−80°C	Not in 3 months	Greengenes	V3–V4 16S rRNA	—	226
4	Lin, 2022, China (Lin et al. 2022)	June 2020‐ December 2020	154 (65:81±10:98)	20 (64:35±12:89)	199 (59:48±3:20)	Self‐sampled	−80°C	Not in 2 months	RDP	V3–V4 16S rRNA	June 2020‐ December 2020	154 (65:81±10:98)
5	Png, 2022, Singapore (Png et al. 2022)	May 2018‐November 2018	12 63.8 (±9.3)	—	25 61.6 (±8.9)	Self‐sampled	−80°C	Not in 3 months	Silva database	V3–V4 16S rRNA	May 2018‐November 2018	12 63.8 (±9.3)
6	Suga, 2022, Japan (Suga et al. 2022)	2017 ‐2020	31	—	10 (56.1±5.7)	Self‐sampled	+4°C	Excluded	RDP	V3–V416S rRNA	2017 ‐2020	31
7	Li, 2020, China (Li et al. 2020)	November 10, 2017 and December 15, 2018	7 59	—	—	Collected at hospital	−80°C	N. D	Greengenes	V 4 − V 6 16S rRNA	November 10, 2017 and December 15, 2018	7 59
8	Yang, 2020, China (Yang et al. 2020)	—	52 53	—	55 42	Collected at hospital	−80°C	Not in 1 month	KEGG	qPCR	—	52 53
9	Park, 2021, South Korea (Park et al. 2021)	August 2017–August 2018	70	—	158	Self‐sampled	−20°C	Not in 3 months	Silva database	V3–V416S rRNA	August 2017–August 2018	70
10	Kasai, 2016, Japan (Kasai et al. 2016)	2012–2013	6 (4) Mean 54	—	6 (4) Mean 50	N. D	+4°C	Current antibiotics excluded	Techno Suruga Lab Microbial Identification Database DB‐BA9.0	V3–V4 16S rRNA	2012–2013	6 (4) Mean 54
11	Berbert, 2022, Brazil (Berbert et al. 2022)	—	28	—	23	Collected at hospital	−80°C	Current antibiotics excluded	Greengenes	V3–V4 16S rRNA	—	28
12	Zorron Cheng Tao Pu, 2019, Australia (Zorron Cheng Tao Pu et al. 2020)	—	25	—	—	Self‐sampled	−80°C	N. D	[not relevant]	V3–V4 16S rRNA	—	25
13	Sánchez‐Alcoholado, 2020, Spain (Sánchez‐Alcoholado et al. 2020)	—	45 62.52±7.99 64.43±7.31	—	20 61.42±7.40	N. D	−80°C	Not in 3 months	Greengenes	V2, 3, 4, and 6–9 of the 16S rRNA	—	45 62.52±7.99 64.43±7.31
14	Shoji, 2021, Japan (Shoji et al. 2021)	June 2018–July 2019	36	—	38	N. D	N. D	Excluded	Greengenes	V2, V4, and V8 or V3, V6–7, and V9 16S rRNA	June 2018–July 2019	36
15	Mira‐Pascual 2016, Spain (Mira‐Pascual et al. 2015)	—	77 71.14 (56–82)	8 (7) 61.75 (41–85)	9 (5) 53.78 (18–69)	N. D	−80°C	N. D	RDP	16S rRNA qPCR	—	77 71.14 (56–82)
16	Gao, 2020, China (Gao et al. 2020)	January 2014 and November 2015 ‐December 2017	155	195	442	Self‐sampled	−80°C	Not in 1 month	RDP	V3–V4 16S rRNA	January 2014 and November 2015 ‐December 2017	155
17	Gao, 2021, China (Gao et al. 2021)	2015‐2018	71	63	91	Self‐sampled	−80°C	Not in 1 month	KEGG	Metagenomic shotgun sequencing	2015‐2018	71
18	Aarnoutse, 2022, Netherlands (Aarnoutse et al. 2022)	March 2017–September 2019	33	—	—	Self‐sampled	at ‐20°C first and at ‐80°C for long‐term	Not in 3 months	[not relevant]	V4 16S rRNA	March 2017–September 2019	33
19	Han, 2019, China (Han et al. 2019)	January 2016‐ September 2017	150	—	—	N. D	N. D	Not in 2 months	N. D	N. D	January 2016‐ September 2017	150
20	He, 2021, China (He et al. 2021)	—	61	—	72	N. D	N. D	N. D	Silva database	V3–V4 16S rRNA	—	61
21	Kikuchi, 2020, Japan (Kikuchi et al. 2020)	April 2017–March 2018		—	—	Self‐sampled	N. D	N. D	Micro SEQ and GreenGenes	V1–V2 16S rRNA	April 2017–March 2018	
22	Yang, 2019, Taiwan (Yang et al. 2019)	2014–2016	62 61.00±10.07	117 60.96±10.09	104 60.71±10.44	Self‐sampled	−80°C	Not in 2 months	Silva database	V3–V4 16S rRNA	2014–2016	62 61.00±10.07
23	Bamola, 2022, India (Bamola et al. 2022)	—	12	—	12	Self‐sampled	−80°C	Not in 1 month	[not relevant]	V316S rRNA	—	12
24	Sarhadi, 2020, Finland (Sarhadi et al. 2020)	April 2015–May 2017	52 62.4±10.6	—	47 62.8±10.1	Self‐sampled	−80°C	N. D	[not relevant]	V2, V4, V8 and V3, V6‐7, V9 of 16S rRNA	April 2015–May 2017	52 62.4±10.6
25	Vogtmann, 2016, USA (Vogtmann et al. 2016)	2004–2006	53	42	61	Self‐sampled	−40°C	N. D	KEGG	whole‐genome shotgun sequencing	2004–2006	53
26	Tarallo, 2019, Italy (Tarallo et al. 2019)	—	29	27	24	Self‐sampled	−80°C	Excluded	Sequence Read Archive (SRA	shotgun sequencing	—	29
27	Feng, 2015, Austria (Feng et al. 2015)	2010–2012	46 (28) 67.1 (43–86)	47 (23) 66.5 (48–84)	63 (37) 67.1 (43–78)	N. D	‐20°C for 48 h, −80°C	Not in 3 months	IMG	16S rRNA	2010–2012	46 (28) 67.1 (43–86)
28	Abbas, 2022, Switzerland (Abbas et al. 2022)	March, 2019‐September 2019	27 63.6 (56.4–76.3)	—	—	Self‐sampled	−80°C	30 days preceding the colon surgery	[not relevant]	V3–V4 16S rRNA	March, 2019‐September 2019	27 63.6 (56.4–76.3)
29	Zhang, 2021, China (Zhang et al. 2021)	—	34 65	—	—	Collected at hospital	−80°C	Not in 3 months	RDP	V3–V4 16S rRNA	—	34 65
30	Jin, 2019, China (Jin et al. 2019)	May 2016 ‐December 2017	15 63(54–83)	23 56.5(22–70)	31 47(23–64)	Collected at hospital	−80°C	Not in 3 months	GreenGenes	V3–V4 16S rRNA	May 2016 ‐December 2017	15 63(54–83)
31	Hua, 2022, China (Hua et al. 2022)	—	154 65.81±10.98	20 64.35±12.89	199 59.48±3.20	Collected at hospital	−80°C	Not in 2 months	Silva database	V3–V4 16S rRNA	—	154 65.81±10.98
32	Yuan, 2022, China (Yuan et al. 2022)	January–December 2020	44 65 Range: 54–84	—	20	Self‐sampled	−80°C	Not in 1 month	SAM tools and Picard	V3–V4 16S rRNA	January–December 2020	44 65 Range: 54–84
33	Yang, 2021, China (Yang et al. 2021)	2018–2021	185 yCRC, 379 oCRC	—	217(yControl) 257(oControl)	Collected at hospital	−80°C	Not in 1 months	Silva database	V3–V4 16S rRNA	2018–2021	185 yCRC, 379 oCRC
34	Zhang, 2018, China (Zhang et al. 2018)	2014‐2015	130 60.5 (9.8)	88 59.6 (10.3)	130 58.6 (8.9)	Collected at hospital	−80°C	Not in 6 months	RDP	V3–V4 16S rRNA	2014‐2015	130 60.5 (9.8)
35	Liu, 2020, China (Liu et al. 2020)	January 2017‐December 2017	51	54	42	Collected at hospital	−80°C	Not in 2 months	Greengene, RDP	V4 16S rRNA	January 2017‐December 2017	51
36	Wang, 2020, China (Wang et al. 2021)	September 2017–August 2018	30 63.9, 6.58	—	30 52.17, 9.02	Collected at hospital	−80°C	Not in 1 month	RDP	16S rRNA high throughput sequencing analysis	September 2017–August 2018	30 63.9, 6.58
37	Shen, 2020, China (Shen et al. 2020)	—	30 58.40±2.36 56.70±6.77	—	25 57.44±2.56	Self‐sampled	−80°C	Excluded	RDP	V3–V4 16S rRNA	—	30 58.40±2.36 56.70±6.77
38	A. Peters, 2016, USA (Peters et al. 2016)	June 2012–August 2014	—	144 63.1±6.6	323 61.3±7.2	Self‐sampled	−80°C	Not in 1 month	IMG/GG Greengenes	V4 16S rRNA	June 2012–August 2014	—
39	Dadkhah, 2019, USA (Dadkhah et al. 2019)	January 2014–June 2015	NR	—	NR	Self‐sampled	‐20°C	N. D	[not relevant]	V1– V2 bacterial 16S rRNA	January 2014–June 2015	NR
40	Wei, 2020, Taiwan (Wei et al. 2020)	—	—	43 56 (43–59)	53 64 (33–69)	Standard containers	−80°C	Not in 3 months	Silva database	16S rRNA	—	—
41	Goedert, 2015, China (Goedert et al. 2015)	—	—	20 (12)	24 (7)	Self‐sampled	−20°C; Moved weekly −80°C	Included	RDP	V3–V4 16S rRNA	—	—
42	Ma, 2024, China (Ma et al. 2024)	October 2021–August 2022	32 (17)	—	—	—	−80°C	Excluded	NR and KEGG	Metagenomic shotgun sequencing	October 2021–August 2022	32 (17)
43	Kaźmierczak‐Siedlecka 2025, Poland (Kaźmierczak‐Siedlecka et al. 2025)	2021–2024	9			Self‐sampled	−80°C	N. D	[not relevant]	NGS sequencing	2021–2024	9

Abbreviations: CRC: colorectal cancer; CRC patients with adverse events in neoadjuvant chemo radiotherapy; ND: not determinate; Non‐AE group: CRC patients with adverse events in neoadjuvant chemo radiotherapy.

**Figure 2:**
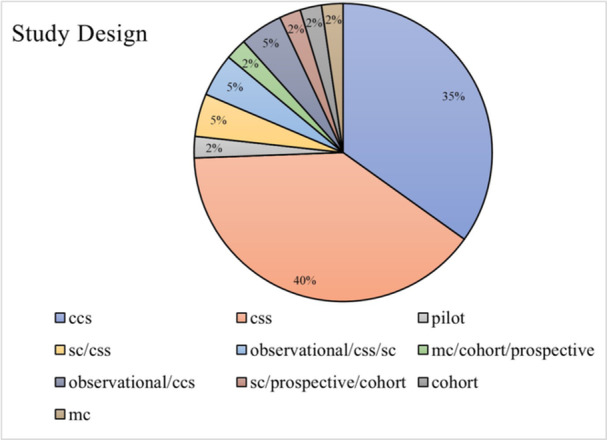
Study type of existing literature. CCS, case‐control study; CSS, cross‐sectional study; MC, multicenter; SC, single center.

The study selection process is presented in Figure 2. The initial database search identified 7573 records. After removal of duplicates, 6248 records remained for title and abstract screening, of which 43 articles were ultimately included.

The included studies were published between 2006 and 2026, with the highest number published in 2021 (21%). China contributed the highest number of studies (*n* = 19). In 25 studies, fecal samples and associated data had been collected between 2006 and 2021, with a reported interval of 2–10 years between sample collection and publication.

The number of fecal samples used for microbiome analysis varied considerably across studies. The smallest studies, by Li et al. and Mira‐Pascual et al. each included seven samples, whereas the largest study, by Yang et al. included 564 samples (Li et al. 2020; Mira‐Pascual et al. 2015; Yang et al. 2021). Among studies reporting CRC cases, the smallest study, by Kasai et al. included six CRC cases, while the largest, by Yuan et al. included 226 CRC cases (Yuan et al. 2021; Kasai et al. 2016).

Pre‐analytical procedures also differed across studies. Most reports described storing stool samples under frozen conditions, although storage protocols were not uniform; only Suga et al. and Kasai et al. reported storage at 4°C (Suga et al. 2022; Kasai et al. 2016). Eligibility criteria related to antibiotic exposure were similarly heterogeneous. Nine studies identified missing or unavailable antibiotic‐use information as a limitation, and one study did not account for antibiotic exposure in participant selection. In the remaining studies, recent antibiotic use was generally an exclusion criterion, although the exclusion window varied from current use to 6 months before recruitment. These differences in sample handling and participant selection are detailed in Table 1.

### 3.2 Microbiome Detection Methods and Analytical Approaches

Culture‐independent techniques have been employed to analyze microbial communities and characterize microorganisms present in fecal samples. These methods rely on variations in the sequence of small subunit ribosomal RNA (16S rRNA) or other target gene regions. Some of the commonly used culture‐independent methods include quantitative real‐time PCR (qPCR), next‐generation sequencing (NGS), Shotgun Metagenomic Sequencing, whole‐genome shotgun sequencing, terminal restriction fragment length polymorphism (T‐RFLP), and high‐throughput sequencing.

In 16S rRNA gene sequencing studies, various variable regions were employed. Specifically, three studies used V1‐V2, nineteen studies used V3‐V4, four studies used V4, one study used V4‐V6, one study used V2, 3, 4, and 6–9, two studies used V2, V4, and V8 or V3, V6–7, and V9, and two studies did not specify the variable region sequenced. 16S rRNA gene sequencing can identify bacterial genera, but it lacks resolution at the strain level and cannot distinguish intraspecies diversity. However, 16S rRNA gene sequencing is still valuable as it provides a starting point by identifying the most common genera, which can be further explored using highly sensitive methods like qRT‐PCR and RNA/whole‐genome sequencing (Whelan and Surette 2017).

The investigations employed diverse reference databases for taxonomic assignment and various statistical approaches to assess potential distinctions among individuals with CRC, adenoma, or healthy controls in terms of their gut microbial community characteristics. Non‐parametric statistical tests and machine learning techniques were utilized.

### 3.3 Fecal Microbiome Compositional Changes in CRC, Adenoma, and Controls

A comparative assessment of fecal microbiome composition across individuals with CRC, adenoma, and healthy controls showed substantial variation at different taxonomic levels. At the phylum level, 12 phyla were reported to vary across the included studies, including *Actinobacteria, Bacteroidetes, Firmicutes, Fusobacteria, Proteobacteria, Verrucomicrobia*, and *Thermodesulfobacteriota* when comparing CRC, adenoma, and controls. The most frequently reported phyla in the studies were *Firmicutes* (97.4%), *Bacteroidetes* (69.2%), *Fusobacteria* (43.5%), *Proteobacteria* (41%), *Actinobacteria*, and *Bacillota* (33.3%). Twenty‐seven studies showed a trend of increased Firmicutes in CRC cases, while 16 studies reported an increase in Firmicutes in controls, and 6 studies reported an increase in adenoma.

At the genus level within the Firmicutes phylum, the taxa most frequently reported in CRC were *Streptococcus, Faecalibacterium, Ruminococcus, Parvimonas, Peptostreptococcus*, and *Blautia*. In controls, the most frequently reported genera within this phylum were *Faecalibacterium* and *Ruminococcus*. Another highly reported phylum was *Bacteroidetes*, within which *Porphyromonas* and *Prevotella* showed the highest prevalence in the CRC group.

The included studies did not demonstrate a consistent consensus regarding the specific fecal bacteria that distinguish individuals with CRC or adenoma from healthy controls. Nevertheless, some recurrent taxa were identified across studies. In particular, the genera Fusobacterium and Porphyromonas emerged as the most frequently reported bacteria, appearing in 16 and 10 studies, respectively, with significantly increased abundance in CRC compared with healthy controls.

**Figure 3:**
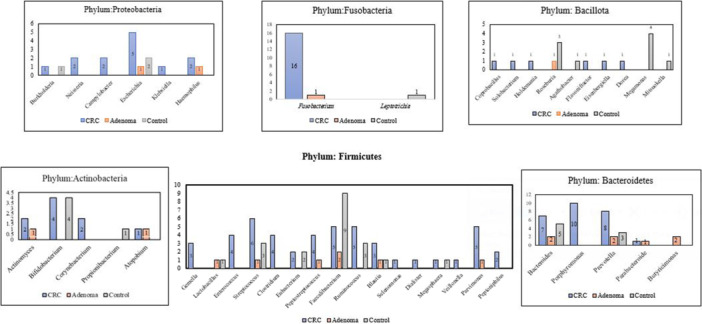
The figure displays the distribution of studies reporting on genera within CRC, adenoma, and control groups. The numbers represent the count of studies within each category.

Further details regarding the abundance of these genera in the three groups (CRC, adenoma, and control) are provided in Figure 3 and Table 2.

**Table 2:** Increased abundance of bacteria in Colorectal Cancer (CRC), Adenoma, and Control Groups.

Taxonomy					Described as being more prevalent in:		
Phylum	Class	Order	Family	Genus	CRC	Adenoma	Control
Actinobacteria					(26)		(26)
	Actinobacteria	Actinomycetales	Actinomycetaceae	*Actinomyces*	(31, 59)	(31)	
		Bifidobacteriales	Bifidobacteriaceae	*Bifidobacterium*	(28, 45, 53, 58)		(24, 36, 38, 48)
		Corynebacteriales	Corynebacteriaceae	*Corynebacterium*	(33)		
		Propionibacteriales	Propionibacteriaceae	*Propionibacterium*	(59)		(62)
	Coriobacteriia	Coriobacteriales	Atopobiaceae	*Atopobium*	(31)	(31)	
Bacteroidetes					(26, 30, 63, 66)		(22, 26, 41)
	Bacteroidia	Bacteroidales	Bacteroidaceae	*Bacteroides*	(25, 38, 42, 53, 56, 60, 63)	(25, 48)	(24, 25, 46, 56, 61)
			Porphyromonadaceae	*Porphyromonas*	(22, 23, 27, 29, 33, 38, 43, 46, 55, 59)		
			Prevotellaceae	*Prevotella*	(24, 28, 32, 34, 38, 41, 44, 56)	(51, 52)	(25, 56, 61)
			Tannerellaceae	*Parabacteroide*	(58)	(48)	
			Odoribacteraceae	*Butyricimonas*		(52, 62)	
				*Odoribacter*	(54)	(52)	
			Rikenellaceae	*Alistipes*	(47, 63)	(48, 52)	
	Flavobacteria	Flavobacteriales	Flavobacteriaceae	*Capnocytophaga*	(35)		
				*Polaribacter*	(35)		
Firmicutes					(22, 26, 63)		(26, 41)
	Bacilli	Bacillales	Bacillaceae	*Bacillus*			
			Bacillales Family XI. Incertae Sedis	*Gemella*	(27, 54, 55)		
		Lactobacillales	Lactobacillaceae	*Lactobacillus*		(51)	(62)
			Streptococcaceae	*Enterococcus*	(33, 34, 43, 58)		
				*Streptococcus*	(27, 34, 37, 40, 54, 59, 63)	(51)	(44, 48, 62)
	Clostridia	Clostridiales	Clostridiaceae	*Clostridium*	(22, 34, 45, 55, 63, 64)		
			Clostridiales Family XIII. Incertae Sedis	*Mogibacterium*			
			Eubacteriaceae	*Eubacterium*	(42, 44)		(37, 38)
			Peptostreptococcaceae	*Peptostreptococcus*	(23, 37, 50, 55)	(52)	
			Peptococcaceae		(27)		
			Ruminococcaceae	*Faecalibacterium*	(25, 32, 42, 53, 56)	(25, 47)	(24, 25, 34, 36‐38, 51, 53, 56)
				*Ruminococcus*	(22, 27, 42, 45, 55)		(32, 48, 51)
				*Blautia*	(60) (36, 58)	(25)	(61)
	Negativicutes	Selenomonadales	Selenomonadaceae	*Selenomonas*	(22)		
		Veillonellales	Veillonellaceae	*Dialister*	(55, 63)		
				*Megasphaera*	(53)		(44)
				*Veillonella*	(44, 63)		
	Tissierellaia	Tissierellales	Peptoniphilaceae	*Parvimonas*	(23, 27, 37, 38, 55)	(52)	
				*Peptoniphilus*	(29, 59)		
Fusobacteria					(22)		
	Fusobacteria	Fusobacteriales	Fusobacteraceae	*Fusobacterium*	(22, 23, 25, 31, 32, 36‐38, 43, 46, 50, 54, 55, 57, 58, 61, 63, 64)	(31)	
			Leptotrichiaceae	*Leptotrichia*			(62)
Proteobacteria							
	Betaproteobacteria	Burkholderiales	Burkholderiaceae	*Burkholderia*	(29)		(62)
				*Ralstonia*			
		Neisseriales	Neisseriaceae	*Kingella*			
				*Neisseria*	(33, 58)		
	Epsilonproteobacteria	Campylobacterales	Campylobacteraceae	*Campylobacter*	(55, 58)		
			Helicobacteraceae	*Helicobacter*			
	Gammaproteobacteria	Enterobacterales	Enterobacteriaceae	*Escherichia*	(34, 38, 40, 41, 56)	(48)	(47, 56)
				*Klebsiella*	(61)		
		Pasteurellales	Pasteurellaceae	*Haemophilus*	(31, 59)	(31)	
Verrucomicrobia					(26)		(26)
Thermodesulfobacteriota	Desulfovibrio desulfuricans	Desulfovibrionales	Desulfovibrionaceae	*Desulfovibrio*	(27, 34)		
				*Bilophila*	(64)	(48)	
Bacillota	Erysipelotrichia	Erysipelotrichales	Coprobacillaceae	*Coprobacillus*	(29, 64)		
			Erysipelotrichidae	*Solobacterium*	(55)		
				*Holdemania*	(58, 64)		
	Clostridia	Eubacteriales	Lachnospiraceae	*Roseburia*		(48)	(37, 38, 56)
				*Agathobacter*			(53)
				*Flavonifractor*	(54)		
				*Eisenbergiella*	(55)		
				*Dorea*	(59)		
	Negativicutes	Selenomonadales	Selenomonadaceae	*Megamonas*			(32, 44, 56, 62)
				*Mitsuokella*			(44)
Pseudomonadota	Alphaproteobacteria	Rhodobacterales	Rhodobacteraceae	*Paracoccus*	(29)		
	Betaproteobacteria	Burkholderiales	Sutterellaceae	*Sutterella*	(56)		
	Gammaproteobacteria	Enterobacterales	Enterobacteriaceae	*Citrobacter*	(38)		
			Morganellaceae	*Morganella*	(43)		
		Aeromonadales	Aeromonadaceae	*Aeromonas*	(43)		(62)
Cyanobacteria	Cyanophyceae	Synechococcales	Synechococcaceae	*Synechococcus*	(29)		
Actinomycetota	Coriobacteriia	Eggerthellales	Eggerthellaceae	*Slackia*			(31)
				*Eggerthella*	(55)		
		Coriobacteriales	Coriobacteriaceae	*Collinsella*	(58)		(53)
Euryarchaeota	Methanobacteria	Methanobacteriales	Methanobacteriaceae	*Methanobrevibacter*	(36)		

### 3.4 Multi‐Bacteria Models for Early Detection of CRC or Adenoma

Three studies incorporated predictive models involving multiple bacteria, which could serve as a potential adjunct to established tests like Guaiac fecal occult blood tests (gFOBT) or fecal immunochemical test (FIT) for non‐invasive early detection of CRC and its precursors. T. Baxter et al. created a cross‐validated random forest model that integrated both FIT and the microbiota for the detection of colonic lesions (Baxter et al. 2016). To discriminate any lesion from normal, the area under curve (AUC) for the multitarget microbiota test (MMT) was notably higher than FIT (MMT AUC: 0.829, FIT AUC: 0.749, *p* < 0.001). When considering specific lesions, the MMT exhibited significantly better performance in detecting adenomas (AUC: 0.755) compared to FIT (AUC: 0.639, *p* < 0.001). However, there was no significant difference in distinguishing cancer from normal (MMT AUC: 0.952, FIT AUC: 0.929, *p* = 0.09).

The random forest model based on microbiota identified 91.7% of cancers and 45.5% of adenomas, surpassing the performance of FIT alone, which detected 75.0% of cancers and 15.7% of adenomas. When FIT missed colonic lesions, the model successfully detected 70.0% of cancers and 37.7% of adenomas. Notably, their findings validated established associations of *Porphyromonas assaccharolytica*, *P. stomatis*, *P. micra*, and *F. nucleatum* with CRC. Additionally, it was observed that the reduction of potentially beneficial organisms, such as those from the Lachnospiraceae family, proved more indicative in identifying patients with adenomas when used in conjunction with FIT. Gao et al. (2021), compared the performance of a microbial panel and FIT for detecting precancer and cancer in colorectal lesions. The microbial panel showed an area under the receiver operating characteristic curve (AUROC) of 0.616 for precancer, with 39% specificity and 83.6% sensitivity, while FIT had an AUROC of 0.682 with 100% specificity and 36.4% sensitivity. Combining the microbiome and FIT increased AUROC to 0.717. For CRC, the 18‐genus panel achieved an AUROC of 0.858, specificity of 98%, and sensitivity of 66.7%, while FIT had an AUROC of 0.978, specificity of 100%, and sensitivity of 95.6%. Integrating the microbiome and FIT further improved performance (AUROC 0.992). The combined model significantly outperformed individual tests, especially for predicting precancer or cancer. Decision curve analysis supported the clinical application of the combined microbial and FIT model.

Yang et al (Yang et al. 2019), compared the performance of gut microbiota biomarkers with conventional non‐invasive methods, such as the non‐quantitative FOBT, serum carcinoembryonic antigen (CEA), and serum carbohydrate antigen 19‐9 (CA19‐9), in detecting and distinguishing young‐onset colorectal cancer (yCRC) and older‐onset colorectal cancer (oCRC). In the Fudan cohort, the microbial model outperformed individual markers and their combinations, achieving AUCs of 0.8981 for yCRC and 0.8511 for oCRC. Validation in the Huadong cohort also demonstrated the superiority of the microbial model, with AUCs of 0.8656 for oCRC and 0.8561 for yCRC, compared to traditional markers. These findings suggest the potential of gut microbiota biomarkers as a promising non‐invasive tool for detecting and distinguishing CRC in different age groups.

## 4 Discussion

CRC poses a significant worldwide health challenge due to its widespread occurrence and high mortality rates. The etiology of CRC is intricate and comprises genetic and environmental factors. The gut microbiota has a significant impact on the initiation, progression, and spread of CRC. With the slow growth of CRC in patients, early diagnosis and population screening offer the possibility of preventing CRC and reducing mortality, which is more effective than in many other cancers (Coker et al. 2019; Nakatsu et al. 2015). This systematic review included 43 studies that examined the variation in gut microbial community composition between individuals diagnosed with CRC or adenoma and those who were asymptomatic, based on human fecal samples.

In this study, we found that the diversity in 12 phyla was markedly altered between individuals with adenoma, CRC, and controls. Six phyla—Firmicutes, Bacteroidetes, Fusobacteria, Proteobacteria, Actinobacteria, and Bacillota were found to be the predominant bacteria in CRC patients, CRC post‐surgery patients, and normal controls. This finding is consistent with the results of studies conducted in Austria, the United States, and the Netherlands (Aarnoutse et al. 2022; Vogtmann et al. 2016; Feng et al. 2015). In this systematic review, multiple researchers found statistically significant disparities in the presence of specific bacteria among individuals with CRC and adenoma compared to healthy individuals. Moreover, within both the CRC and adenocarcinoma cases in this study, the most prevalent bacterial phylum identified was Firmicutes, followed by Bacteroidetes. Consistent with these findings, several studies have also documented an increase in certain bacteria in CRC and adenoma (Pandey et al. 2023). The phyla of bacteria frequently linked to colorectal carcinogenesis and adenocarcinoma include Firmicutes (Allali et al. 2018; Baxter et al. 2016; Yang et al. 2020; Kasai et al. 2016; Berbert et al. 2022; Sánchez‐Alcoholado et al. 2020; Gao et al. 2020; Aarnoutse et al. 2022; Kikuchi et al. 2020; Sarhadi et al. 2020; Abbas et al. 2022; Zhang et al. 2021; Jin et al. 2019; Wang et al. 2021; Peters et al. 2016), Bacteroidetes (Allali et al. 2018; Lin et al. 2022; Park et al. 2021; Kasai et al. 2016; Berbert et al. 2022; Zorron Cheng Tao Pu et al. 2020; He et al. 2021; Kikuchi et al. 2020; Bamola et al. 2022; Feng et al. 2015; Liu et al. 2020; Alomair et al. 2018; Ren et al. 2020) and both Firmicutes and Bacteroidetes (Aarnoutse et al. 2022; Wang et al. 2021). These findings suggest that phylum‐level alterations in CRC do not simply describe which bacteria are present; they may represent several overlapping biological processes. Many health‐associated members of the Firmicutes phylum, such as butyrate‐producing taxa, contribute to epithelial energy supply, maintenance of barrier integrity, and anti‐inflammatory immune regulation through short‐chain fatty acid (SCFA) production (Louis and Flint 2017; Parada Venegas et al. 2019). Therefore, phylum‐level preservation or enrichment of Firmicutes does not exclude the possibility that key SCFA‐producing commensals are selectively depleted in CRC and the reported shift in Firmicutes may represent a restructuring of this phylum away from beneficial butyrate producers and toward taxa less capable of maintaining mucosal homeostasis. Likewise, members of Bacteroidetes phylum are central to complex carbohydrate fermentation, but some taxa are also involved in mucin utilization and inflammatory signaling. This is particularly relevant because disruption of the mucus barrier may increase epithelial exposure to microbial products and facilitate tumor‐promoting inflammation (Derrien et al. 2017; Tailford et al. 2015). In addition, specific strains such as enterotoxigenic Bacteroides fragilis have been shown to promote colorectal tumorigenesis through toxin‐mediated epithelial injury and activation of pro‐carcinogenic immune pathways, including Th17‐related inflammation (Wu et al. 2009). Thus, increased representation of Bacteroidetes may reflect not only altered carbohydrate metabolism but also mucosal barrier stress and selective enrichment of pro‐inflammatory organisms. Also, the fact that Firmicutes and Bacteroidetes remain dominant in CRC shows that CRC‐associated dysbiosis appears to involve functional remodeling of the gut ecosystem rather than replacement of the dominant phyla altogether (Janney et al. 2020; Wong and Yu 2019). Similarly, increased Proteobacteria may indicate an inflamed and metabolically disturbed intestinal environment and expansion of Enterobacteriaceae, which is widely regarded as a marker of ecological instability and inflammation, because these organisms can thrive under conditions of increased oxygen tension, nitrate availability, and oxidative stress (Arthur et al. 2012; Ridlon et al. 2014). In the CRC setting, this pattern may also represent enrichment of pathobionts with carcinogenic potential. For example, certain Escherichia coli strains carrying the polyketide synthase island produce colibactin, a genotoxin capable of inducing DNA damage and mutational signatures identified in human CRC (Pleguezuelos‐Manzano et al. 2020). Thus, a Proteobacteria‐rich signal may indicate a microenvironment favorable to inflammation‐associated pathobiont expansion and genotoxic stress. Overall, these phylum‐level alterations like loss of SCFA‐mediated colonization resistance and epithelial support, expansion of oral‐derived or inflammation‐adapted organisms, increased mucin degradation and barrier perturbation, and microbial metabolic shifts that alter bile‐acid transformation and other luminal metabolites are linked to colorectal carcinogenesis (Louis and Flint 2017; Tailford et al. 2015; Wong and Yu 2019; Ocvirk and O'Keefe 2017).

Furthermore, earlier research, which aligns with our findings, has documented *Streptococcus, Faecalibacterium*, and *Ruminococcus* as the predominant genera within the Firmicutes phylum (Yang et al. 2020; He et al. 2021; Kikuchi et al. 2020; Sarhadi et al. 2020; Vogtmann et al. 2016; Abbas et al. 2022; Jin et al. 2019) while *Porphyrmonas* (Allali et al. 2018; Yang et al. 2020; Vogtmann et al. 2016; Abbas et al. 2022) and *Prevotella* (Kasai et al. 2016; Sánchez‐Alcoholado et al. 2020; Mira‐Pascual et al. 2015; He et al. 2021; Liu et al. 2020) were the most frequently identified genera in the CRC group. Moreover, a number of recent reviews suggest that there is a positive correlation between the abundance of *Fusobacterium* and the progression to more advanced stages of cancer (Amitay et al. 2018; Ternes et al. 2020). Within Firmicutes, Faecalibacterium is widely regarded as a major butyrate‐producing and potentially protective commensal (Smith et al. 2013; Donohoe et al. 2014; Sokol et al. 2008). Butyrate contributes to epithelial barrier maintenance, immune regulation, and suppression of colorectal tumorigenesis; therefore, depletion of Faecalibacterium has been interpreted as a marker of loss of protective SCFA‐producing capacity. By contrast Streptococcus may reflect a different process, including oralization of the gut microbiome, altered luminal redox conditions, or a more inflammatory mucosal environment (Koliarakis et al. 2019). Similarly, Porphyromonas may support the concept of oral microbial translocation or oralization of the colonic ecosystem, a phenomenon increasingly linked to biofilm formation, local inflammation, and tumor‐associated dysbiosis (Koliarakis et al. 2019; Flemer et al. 2017). In the current review, which encompasses most of the studies, there were no significant differences in the relative abundance of *Fusobacterium* or any other bacteria between participants with CRC or adenoma and healthy participants. This inconsistency may arise from differences in tumor stage, anatomic location, antibiotic exposure, bowel preparation, DNA extraction methods, sequencing platforms, and geographic or dietary variation across cohorts (Zeller et al. 2014). Taken together, our findings suggest that the microbial patterns observed in fecal samples from patients with adenoma and CRC are consistent with a functionally disturbed ecosystem.

Surgical stress induces physiological changes in the human body, which, in turn, elicit responses from the host that affect the intestinal microenvironment. This disturbance in the delicate ecological balance of the gut is a consequence of these interactions. In the present research, after undergoing colorectal cancer surgery, patients exhibited a significant rise in the levels of Actinobacteria and Firmicutes, which is similar to the finding reported by Liu et al. in the Department of Surgery in China (2020) (Liu et al. 2020), and Ohigashi et al. in the Department of Gastroenterological Surgery in Tokyo (2013) (Ohigashi et al. 2013). Chin Wen Png also found an overabundance of Actinobacteria and Firmicutes, while some Bacteroidetes were reduced in post‐operation CRC patients compared to pre‐operation patients (Png et al. 2022). Recent studies by Deng et al (Deng et al. 2018)., Yao et al (Yao et al. 2021). and Kong et al (Kong et al. 2019). showed decreased numbers of Firmicutes and Bacteroidetes but an increase in Proteobacteria in patients after CRC surgery compared to healthy individuals or CRC patients. The discrepancy between different study results could be due to a small sample size, inefficient culture through conventional methods for bacterial identification, and differences in the regions of the colon in CRC patients, which can lead to variations in the results of the mentioned studies. It is of great importance to identify the differences between left‐sided colon cancer (LCC) and right‐sided colon cancer (RCC), including variations in symptoms, molecular characteristics, and treatment response, to discover more effective markers that can guide future clinical research. Our findings align with previous research, demonstrating apparent dissimilarities in the microbiome composition between patients with tumors located in the left and right colon (Flemer et al. 2017). Additionally, the findings from this research demonstrate that microbiota in LCC samples, such as *Bacteroides* and *Fusobacterium*, could potentially contribute to the development of colon cancer, while the microbiota in RCC samples, particularly *Bacteroides*, poses a detrimental risk. In Suga D's research, it was indicated that the RCC group had a higher prevalence of Firmicutes, and the LCC group had a higher abundance of *Verrucomicrobia*. At the genus level, *Ruminococcaceae*, *Streptococcaceae*, *Clostridiaceae*, *Gemellaceae*, and *Desulfovibrio* were found in the RCC group, and several oral microbiomes were observed in the LCC group (Suga et al. 2022). Overall, these findings support the concept that the relative enrichment of *Fusobacterium* in LCC may indicate a tumor‐associated microbial, because *Fusobacterium nucleatum* has been linked to epithelial adhesion, activation of inflammatory and β‐catenin signaling pathways, modulation of the tumor immune microenvironment, and resistance to chemotherapy in colorectal cancer (Kostic et al. 2013; Rubinstein et al. 2013; Brennan and Garrett 2019). By contrast, *Bacteroides* genus includes both common commensals and strains with pathogenic potential Therefore, an increased abundance of *Bacteroides* in either LCC or RCC may better reflect altered mucosal ecology or anaerobic niche selection (Wong and Yu 2019).

T. Baxter et al. found that the integration of microbiota analysis with FIT enhances the performance of colonic lesion detection. Importantly, the study confirmed the known associations of specific microbial species with CRC, such as *Porphyromonas assaccharolytica, P. stomatis, P. micra, and F. nucleatum*. Additionally, the reduction of potentially beneficial organisms from the *Lachnospiraceae* family proved to be indicative in identifying patients with adenomas, especially when used alongside FIT. Furthermore, the findings of two recent studies displayed the potential of gut microbiota biomarkers as a non‐invasive technique to detect and differentiate colorectal cancer in both young‐onset and older‐onset individuals.

Nonetheless, as observed in the current study, no consensus has been reached regarding the specific fecal bacteria that distinguish the gut microbiome of individuals with CRC or adenoma from healthy controls. However, several studies in this review consistently highlight the genus *Fusobacterium* and *Porphyromonas* as frequently reported bacteria in the majority of the examined research. These bacteria exhibit a significant increase in abundance among CRC cases compared to healthy controls. Further research is needed to comprehensively understand the role of these bacteria in the development and progression of colorectal cancer. Certain factors, such as differences in samples, collection methods, storage temperatures, interval preceding the microbiome analysis of samples, processing methods (RT‐PCR or high‐throughput analyses), and the stage and location of tumors, may influence effect of gut microbiota on CRC.

In addition, the findings of our study align with previous research, which reported that the composition and relative abundance of the gut microbiome are influenced by age. There is a general pattern of increased diversity from childhood to adulthood, followed by a subsequent decline in older age groups (over 70 years) (Zhang et al. 2021; Ishibashi et al. 2019; Odamaki et al. 2018). Important findings about the importance of microbial composition in CRC, were achieved in this research; however, shortcomings and restrictions persisted. Several factors may affect the consistency of results of the reviewed studies on the effect of gut microbiota on CRC, including differences in the type of samples, collection methods, storage temperature, and time interval before microbiome analysis, processing method, different tumor stages or location can affect the diversity. In addition, the article focuses on specific studies and findings related to the gut microbiome in CRC patients, limiting the generalizability of the results to broader populations or clinical settings. Overall, the article provides valuable insights into the role of the gut microbiome in colorectal cancer and highlights the need for continued research to enhance our understanding of microbial markers for early detection and treatment of CRC.

It is important to note that the choice of the hypervariable region for the 16S rRNA gene and the analysis of the data with diverse reference databases and statistical approaches can impact the analysis and lead to bias in microbial diversity. Additional research is needed to better understand the advanced sequencing techniques such as metatranscriptomic and metagenomic sequencing, may have the potential to enhance early detection and risk assessment of CRC.

## 5 Conclusion

In conclusion, based on the existing data, several intestinal bacteria exhibit changes along the adenoma‐carcinoma sequence, indicating their potential as markers for the diagnosis and treatment of colorectal adenoma/carcinoma. Furthermore, the differences in the fecal gut microbiome between individuals with CRC or adenoma and those without malignancy offer promising but limited evidence for the development of cost‐effective and non‐invasive fecal tests. It is important to take into account host factors when studying the characteristics of the intestinal microbiota in CRC.

## Author Contributions

**Parvin Askari**, **Shirin Dashtbin**, **Tahereh Navidifar**, and **Parisa Najafi:** writing – original draft. **Leila Dadgar‐Zankbar:** writing – review and editing. **Arezoo Asadi:** writing – review and editing. **Mahsa Ghamari:** investigation. **Shabnam Zeighamy Alamdary:** investigation. **Roghayeh Afifirad:** investigation. **Roya Ghanavati:** conceptualization, funding acquisition. **Atieh Darbandi:** conceptualization.

## Ethics Statement

This study was approved by the Ethics Committee (code number: IR.BHN.REC.1402.015) of Behbahan Faculty of Medical Sciences.

## Consent

The authors have nothing to report.

## Conflicts of Interest

The authors declare no conflicts of interest.

## Data Availability

All relevant data are included in the manuscript. The data that support the findings of this study are available on request from the corresponding author. The data are not publicly available due to privacy or ethical restrictions.
